# The pathway to resolve dimeric forms distinguishes plasmids from megaplasmids in Enterobacteriaceae

**DOI:** 10.1093/nar/gkae1300

**Published:** 2025-01-11

**Authors:** Florian Fournes, Manuel Campos, Jean Cury, Caroline Schiavon, Carine Pagès, Marie Touchon, Eduardo P C Rocha, Philippe Rousseau, François Cornet

**Affiliations:** Laboratoire de Microbiologie et de Génétique Moléculaires, Centre de Biologie Intégrative, Université de Toulouse, CNRS, 165 Rue Marianne Grunberg-Manago, campus Paul Sabatier, 118, route de Narbonne, 31062, Toulouse Cedex, France; Laboratoire de Microbiologie et de Génétique Moléculaires, Centre de Biologie Intégrative, Université de Toulouse, CNRS, 165 Rue Marianne Grunberg-Manago, campus Paul Sabatier, 118, route de Narbonne, 31062, Toulouse Cedex, France; Microbial Evolutionary Genomics, Institut Pasteur, CNRS, 25-28 Rue du Dr Roux, 75015 Paris Cedex, Paris, France; Laboratoire de Microbiologie et de Génétique Moléculaires, Centre de Biologie Intégrative, Université de Toulouse, CNRS, 165 Rue Marianne Grunberg-Manago, campus Paul Sabatier, 118, route de Narbonne, 31062, Toulouse Cedex, France; Laboratoire de Microbiologie et de Génétique Moléculaires, Centre de Biologie Intégrative, Université de Toulouse, CNRS, 165 Rue Marianne Grunberg-Manago, campus Paul Sabatier, 118, route de Narbonne, 31062, Toulouse Cedex, France; Microbial Evolutionary Genomics, Institut Pasteur, CNRS, 25-28 Rue du Dr Roux, 75015 Paris Cedex, Paris, France; Microbial Evolutionary Genomics, Institut Pasteur, CNRS, 25-28 Rue du Dr Roux, 75015 Paris Cedex, Paris, France; Laboratoire de Microbiologie et de Génétique Moléculaires, Centre de Biologie Intégrative, Université de Toulouse, CNRS, 165 Rue Marianne Grunberg-Manago, campus Paul Sabatier, 118, route de Narbonne, 31062, Toulouse Cedex, France; Laboratoire de Microbiologie et de Génétique Moléculaires, Centre de Biologie Intégrative, Université de Toulouse, CNRS, 165 Rue Marianne Grunberg-Manago, campus Paul Sabatier, 118, route de Narbonne, 31062, Toulouse Cedex, France

## Abstract

Bacterial genomes contain a plethora of secondary replicons of divergent size. Circular replicons must carry a system for resolving dimeric forms, resulting from recombination between sister copies. These systems use site-specific recombinases. Among these, the XerCD recombinase resolves dimers of chromosomes and certain plasmids, using different modes of regulation. We have analyzed the dimer resolution functions in enterobacterial secondary replicons and show that, in addition to the main chromosomes, XerCD is preferentially used by small plasmids and by the largest secondary replicons, megaplasmids and secondary chromosomes. Indeed, all replicons longer than 250 kb host an active XerCD recombination site. These sites, in contrast to those of small plasmids, use the same control as chromosomes, coupled to cell division by the FtsK protein. We conclude that a chromosome-like mode of dimer resolution is mandatory for the faithful inheritance of large plasmids and chromids, its acquisition being a prerequisite for the genesis of secondary chromosomes from plasmids.

## Graphical Abstract

**Graphical Abstract:**
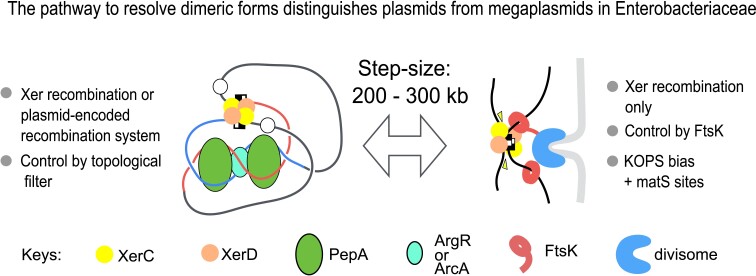

## Introduction

Besides a unique main chromosome, bacterial genomes contain a plethora of secondary replicons differing in fate, composition and size (1–8). These are found in most bacterial genomes and carry adaptive traits along with functions for their own maintenance and dissemination. Most of them are mobile genetic elements that can transfer between strains and species, bringing, for example, the acquisition of symbiosis or pathogenic power and resistance to antibacterial compounds (9–11).

Secondary replicons differ in size, copy number and the maintenance and transfer functions they encode, allowing the definition of replicon categories (1,12). Small ‘high-copy-number’ (HCN) plasmids are <25 kb long. Moderate- to low-copy-number (LCN) plasmids are larger, most of the time about 100 kb in enterobacteria, and frequently encode the complex machinery for their self-transfer by conjugation. The term megaplasmids was proposed for notably large LCN plasmids that show no important chromosome-like feature (5,7). They frequently have mosaic structures, carrying evidence of plasmid co-integration and of extensive gene transfer (13). Lastly, chromids and secondary chromosomes, often referring to the same replicon categories, are large replicons, typically above 500 kb, found in the different strains of a species, thus becoming part of its core genome (6,7,14). They carry core genes and appear more adapted to their host than plasmids and megaplasmids since they display nucleotide composition and codon usage closer from the main chromosome.

**Figure 1.**
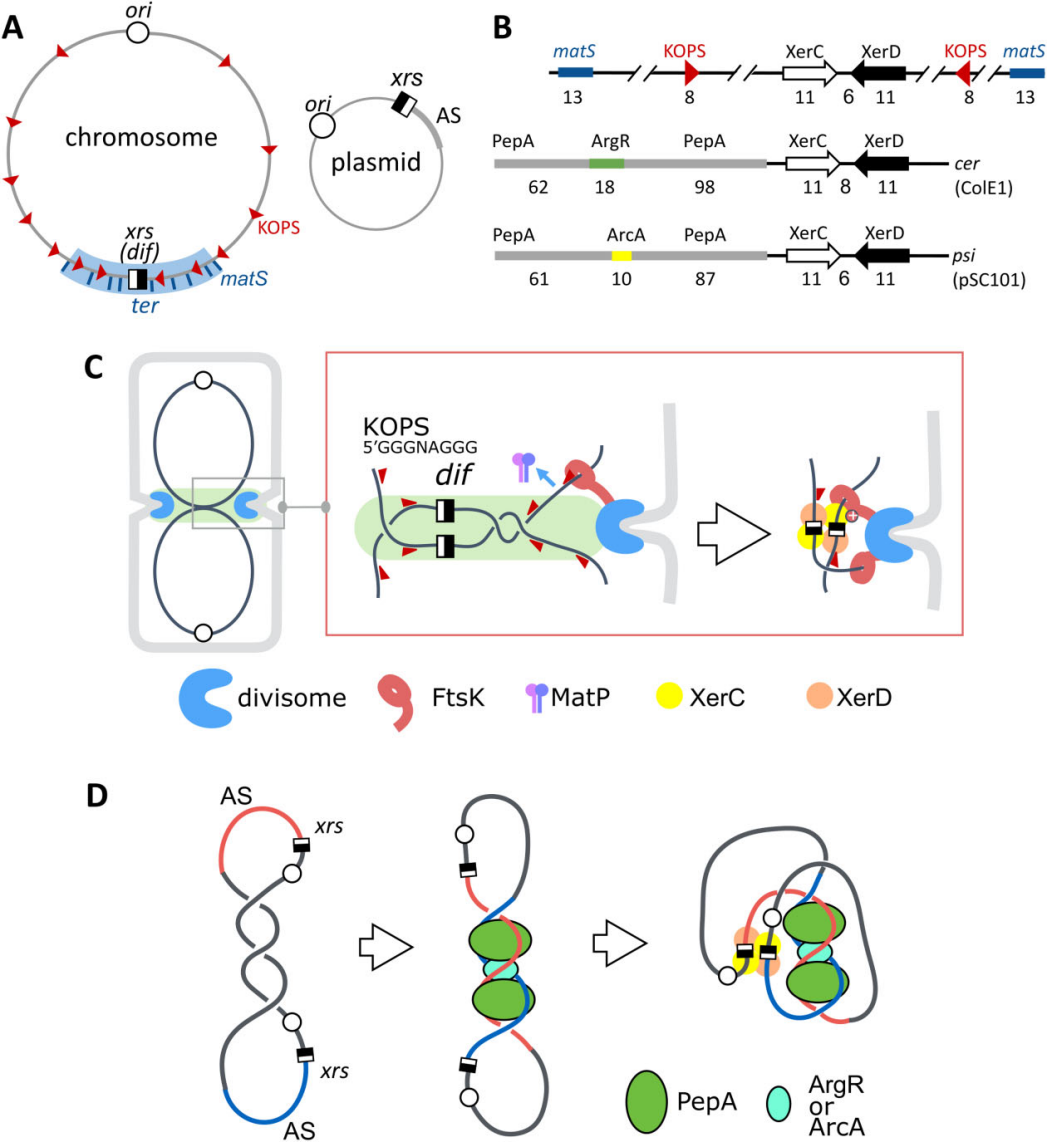
The Xer system and its modes of regulation. (**A**) Replicon maps showing *xrs* (black and white squares) with relevant DNA elements involved in the control of recombination: KOPS (arrowheads) and *matS* in the case of chromosomes (left) and accessory regions (AS) in the case of small multicopy plasmids (right). (**B**) Local organization around *xrs* in the case of the chromosome (*dif* site, top line) and the two model plasmids ColE1 (*cer* site, middle) and pSC101 (*psi* site, bottom line). Binding sites for XerC and XerD, ArgR and ArcA are shown. PepA binds with poor specificity in the regions surrounding the ArgR and ArcA binding sites. Sizes of the different elements are indicated in bp. (**C**) In the case of the chromosome, recombination occurs in pre-divisional cells at the onset of cell division (left drawing) and is controlled by the MatP and FtsK proteins. MatP dimers bind to *matS* sites scattered in the *ter* region (Figure 1) and keep it in the mid-cell zone, allowing septum-borne FtsK hexamers to load onto *ter* DNA and translocate DNA toward the *dif* site following the orientation of the KOPS motifs (arrowheads). Upon reaching the XerCD/*dif* complex, FtsK induces recombination, resolving the chromosome dimer to monomers. (**D**) Recombination between *xrs* carried by model small plasmid depends on DNA ASs flanking the XerC binding site. In a plasmid dimer, the two ASs get together by sliding into the plectonems created by negative supercoiling and are recognized by the PepA and either ArcA or ArgR proteins. This creates a structure trapping three DNA crossing, required to induce recombination resolving the dimer. This mechanism ensures that recombination is favored between the *xrs* carried by a dimer.

Circular replicons should contain at least one system to resolve dimeric forms (12,15,16). Dimers or higher multimers arise from homologous recombination between sister copies and lower the number of plasmid copies, hindering stable inheritance. They are resolved by site-specific recombination. Resolution systems have been mainly studied in some model HCN plasmids and in chromosomes. Chromosomes use the XerCD recombinase, activated by the FtsK DNA translocase, to recombine the XerCD recombination site (*xrs*) they carry, called the *dif* site (Figure 1A). *Xrs* are short pseudo-palindromic sequences (28–30 bp) imperfectly conserved, consisting of two 11 bp binding sites for XerC and XerD, respectively, separated by a 6–8 bp central region (Figure 1B). The terminal region of the chromosome (*ter*), where the *dif* site lies, carries numerous *matS* sites recognized by the MatP protein (17). MatP delays *ter* segregation and tethers sister *ter* to the division septum to which the FtsK DNA translocase is linked. FtsK segregates sister *ter* following the orientation dictated by the KOPS motifs, ending at *dif*, and then activates XerCD recombination in case sister chromosomes are dimeric (Figure 1C) (12,18). Some model HCN plasmids, i.e. ColE1 and pSC101, also contain *xrs* and use XerCD. In this case, recombination does not depend on FtsK but requires 200-bp-long DNA sequences (called accessory sequences, ASs) adjacent to the XerC binding site of the *xrs* (Figure 1B). ASs are recognized by specific proteins, either the ArgR (plasmid ColE1 and relatives) or ArcA (plasmid pSC101) and the PepA protein in both cases. These proteins form a nucleoprotein complex containing pairs of ASs only when present on the same molecule, ensuring that recombination resolves dimers better than creating them (Figure 1D) (16). ArgR and ArcA bind cognate sites at defined position in ASs, whereas PepA does not bind recognizable DNA motif but is attracted by either ArgR or ArcA (19).

Here, we asked how the two described control modes of dimer resolution are distributed in secondary replicons. We analyzed enterobacterial secondary replicons for their dimer resolution mode. We show that replicons encode site-specific recombinase and/or possess *xrs*. Replicon size appears as the main determinant for dimer resolution strategy. HCN plasmids tend to harbor *xrs* associated with AS, whereas LCN plasmids tend to encode their own recombinases. Strikingly, the larger replicons show a tendency to possess *xrs* that increase with their size and become a rule above 250 kb. We analyzed the R27 plasmid and show that it possesses an FtsK-activated *xrs* able to resolve its dimeric forms. A selected array of *xrs* cloned from large replicons also recombined in an FtsK-dependent manner. We further show that KOPS biases and *matS* sites arise on the largest replicons. We conclude that acquiring FtsK-controlled dimer resolution is compulsory for replicons to grow significantly over the size of LCN plasmids.

## Materials and methods

### Strains, plasmids and standard procedures

DNA and *Escherichia coli* strain manipulations and constructs all followed standard procedures. Plasmid R27 was a gift from Carlos Balsalobre (University of Barcelona, Spain). From the R27 sequence (20), gene R0101 was identified with the help of Laurence Van Melderen’s laboratory (University of Bruxelles, Belgium) as encoding a potential toxin. It was deleted with the neighbor gene, R0102, and replaced by the *lacI* gene, yielding our ‘wt’ R27. Gene R0183 encodes a potential Y-recombinase and was replaced by an FRT-Kn-FRT cassette subsequently resolved to a single FRT site using the FLP recombinase. A potential *xrs* was identified as the 5′-AGTACATATACCAAAGATTATGTTAAAT sequence. This region was sequenced, allowing to correct the C at position 11 to an A. The corrected *xrs* was subsequently cloned in pUC57 backbone and used. Plasmids for recombination monitoring were constructed using polymerase chain reaction (PCR) assembly to first obtain *xrs*-Kn module, which was then inserted in pUC57 derivatives already containing *xrs*, resulting in *xrs*-Kn-*xrs* cassettes.

Strains used for recombination assays are derivatives of DS941 (21) rendered Δ(*xerC*)::Cm, Δ(*pepA*)::Kn or Δ(*ftsK*_C_)::Tc. Strains were transformed by plasmids carrying directly repeated *xrs*, grown overnight on antibiotic-containing plates. Ten colonies were picked up and grown before plasmid extraction and analysis on 1% agarose 1X tris acetate EDTA (TAE) gels.

R27 loss was measured in derivatives of strain LN2666 (22) first rendered Δ(*lacI*)::Sp, and then either Δ(*xerC*)::Cm, Δ(*xerD*)::Kn or *ftsK^ATP^*^−^-Cm by standard procedures. Strains containing R27 derivatives carrying the *lacI* gene were cultivated overnight in L broth plus tetracycline, then diluted twice per day and grown in serial cultures without tetracycline for 3 days, corresponding to 60 generations, before plating on X-Gal-containing plates. The ratio of blue colonies (R27 loss) on total is reported with standard deviation of three independent experiments.

### Protein purification and *in vitro* reactions

Protein purifications were performed as described in (23) for *E. coli* XerC and XerD recombinases and in (24) for the tαβγFtsK_C_ protein.

For electrophoretic mobility shift assays, radiolabeled 28-bp DNA fragments containing *xrs* were incubated with increasing concentration of XerC and/or XerD proteins (from 0.2 to 0.8 mM), as reported in and visualized using Typhoon-Trio-GE.

*In vitro* recombination experiments were mainly performed as previously reported in (25). Briefly, 300 ng of plasmid DNA containing *xrs*-Kn-*xrs* cassette was incubated with final concentrations of 0.3 mM of XerC and XerD proteins and 0.5 mM of tαβγFtsK_C_, in a buffer containing 25 mM Tris–HCl (pH 7.5), 10 mM MgCl^2^, 0.1% PEG8000 and 2.5 mM ATP, and incubated for 2 h at 30°C. Reactions were stopped using a buffer containing 10% SDS and 2 mg/ml of proteinase K and then analyzed by 0.8% agarose gel electrophoresis.

### *xrs* sites and AS detection

Site-specific recombinases were searched using hidden Markov models (HMM) profiles. To search for *xrs*, we started from the few *xrs* functionally characterized (Supplementary File S1), searched by homology using BLASTN and then checked manually the retrieved sequences applying some rules: (i) the internal parts (first 5 bp) of the XerC and XerD binding sites are the most conserved and often form a palindrome; (ii) the XerD binding site is better conserved than the XerC binding site; and (iii) the central region is 6–8 bp long and its sequence is not conserved. The retrieved *xrs* were added to the search until no additional *xrs* was retrieved. A total of 517 *xrs* were found, of which 185 were unique sequences (Supplementary File S2).

As the previously characterized ASs are poorly conserved, we relied on searching potential ArcA or ArgR binding sites at the same positions as in known AS. Retrieved ASs were then used for homology search and this procedure was repeated until no new AS was retrieved. Most ASs contained an ArgR binding site, only four harboring one for PepA. The most divergent examples are shown in Supplementary File S1.

### KOPS detection

Strategies and scripts for KOPS and *matS* detection and analysis are described at https://doi.org/10.5281/zenodo.14046856. We identified KOPS with the regular expression function from the built-in Python package re, matching the pattern ‘GGG[ACGT]AGGG’ and its reverse complement ‘CCCT[ACGT]CCC’. For representation analysis, we used R’MES (26) to calculate a score for the motif GGGNAGGG with a maximal Markov chain length (*l* = 6) and considered, as recommended, the values of −3 and 3 as threshold for under- and over-represented cases, respectively. For skew analysis, KOPS positions were translated into angles after a circular shift of the plasmid DNA sequence so as to place the *xrs* site at the middle of the sequence (Supplementary Figure S3A and B). The angle of the *i*th instance of the motif, ${\theta _i}$, is calculated as


\begin{eqnarray*}{\theta _i} = 2\pi \frac{{{l_i}}}{L},\end{eqnarray*}

where ${l_i}$ is the position of the *i*th instance of the motif along the re oriented sequence and *L* is the length of the plasmid sequence.

KOPS clustering was evaluated with the resultant length of the set of angles formed by all instances along the plasmid DNA sequence. The square of the resultant length is defined as


\begin{eqnarray*}{R^2} = {\left( {\frac{1}{N}\mathop \sum \nolimits_{i = 1}^N \cos {\theta _i}} \right)^2} + {\left( {\frac{1}{N}\mathop \sum \nolimits_{i = 1}^N \sin {\theta _i}} \right)^2},\end{eqnarray*}

where ${\theta _i}$ is the angle of the *i*th instance and *N* is the number of instances.

The statistical significance of clustering was estimated using the Rayleigh test included in the circstats module of the Astropy Python package (27). Low KOPS occurrences limit our ability to detect clustering in small plasmids. We estimated the statistical power to fall below 0.5 for plasmids with <15 KOPS occurrences.

### *matS* sites’ detection

The 23 *matS* of the chromosome of *E. coli* K12 MG1655 were used to construct a position-specific score matrix (PSSM; Supplementary Figures S3C and D; https://doi.org/10.5281/zenodo.14046856). The threshold score to defined a site as a bona fide *matS* was defined so as to selectively detect all 23 *matS* sites previously identified on *E. coli* K12 MG1655 chromosome (17). To analyze *matS* representation, 1000 random sequences were generated for each plasmid using a custom Python script (rnseq.py) that extend the DNA sequence by matching their 4-mer distribution of each plasmid. The number of *matS* sites in these random sequences thus represents the expected number of *matS* sites under a Markov model with a Markov chain of length 4. The distribution of *matS* counts in these recoded sequences was effectively approached by a Poisson distribution used to estimate how (un)likely it is to observe the actual *matS* counts in real plasmid sequences as $P( {X = k} ) = {{{\lambda ^k}{{\rm e}^{ - \lambda }}}}/{{k!}}$, with $k = {n_{{\rm observed}}}$ and $\lambda = {\bar{n}_{{\rm random}}}$.

## Results

### Plasmid size determines the presence of *xrs*

**Figure 2.**
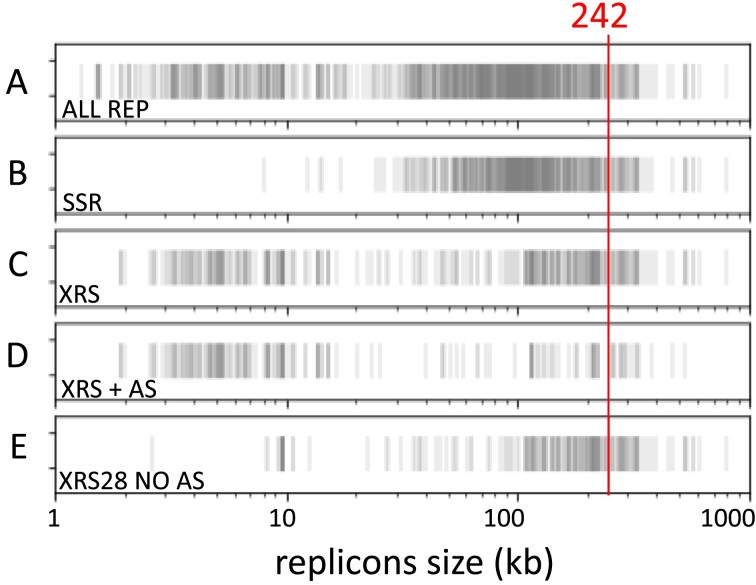
Plasmid size determines the dimer resolution systems used. Eventplots of replicons ranked by their size (*x*-axis): (**A**) all 981 replicons; (**B**) presence of at least one site-specific recombinase; (**C**) presence of at least one potential *xrs*; (**D**) presence of at least one potential *xrs* flanked by a potential AS next to the XerC binding site; and (**E**) presence of at least one potential 28-bp-long *xrs* (6-bp-long central region) devoid of recognizable AS. The size above which all replicons harbor at least one 28-bp-long *xrs* devoid of AS is indicated.

To predict how secondary replicons cope with dimer resolution, we performed a survey of the site-specific recombinases they encode and a search for XerCD recombination sites (*xrs*) they carry (see the ‘Materials and methods’ section). We used a collection of 981 Enterobacteriaceae secondary replicons previously annotated for other maintenance functions (28). These replicons ranged from 1.3 to 794 kb in size and showed a bimodal size distribution: 25% were <25 kb and harbored replication functions suggesting either rolling circle-type replication or theta-type replication with high copy number per cell, while the others were centered around the 50–150 kb range (half of the plasmids) and predicted to be low copy number from their replication functions (Figure 2, lane A). Only 10% of the replicons were larger than 200 kb and 7 larger than 500 kb.

Site-specific recombinases were frequent in plasmids larger than 25 kb and tended to be absent in smaller plasmids (Figure 2, lane B). Small plasmids tended to harbor *xrs* (70% of the plasmids smaller than 25 kb; Figure 2, lane C), while medium-sized plasmids (30–100 kb) tended to have no *xrs*. Strikingly, most plasmids larger than 115 kb and all replicons above 242 kb had at least one *xrs*.

*Xrs* may recombine in an AS- and proteins-dependent manner whatever their size: 28, 29 or 30 bp. In contrast, only 28 bp *xrs* were shown to recombine in an FtsK-dependent manner (16,29,30). Of the eight ASs previously reported, only two pairs displayed weak yet significant homology. We thus relied on the presence of ArgR or ArcA binding sites at the correct distance from the *xrs* (Figure 1A) in addition to homology search of ASs (see the ‘Material and methods’ section; Supplementary File S1). All *xrs* present on small plasmids were associated with an AS, whereas *xrs* present on large replicon tended to be devoid of AS (Figure 2, lane D). In contrast, 28 bp *xrs* not associated with an AS were almost restricted to large replicons (Figure 2, lane E), suggesting that these replicons use FtsK-dependent recombination to resolve dimers. Taken together, our analysis suggests three types of replicons with respect to dimer resolution: small plasmids tend to use XerCD in an AS- and PepA-dependent manner and mid-size plasmids tend to use a self-encoded system, while the largest replicons tend to use XerCD in a FtsK-dependent manner, which becomes a rule above 250 kb.

### The R27 plasmid resolves dimers in an FtsK-dependent manner

**Figure 3.**
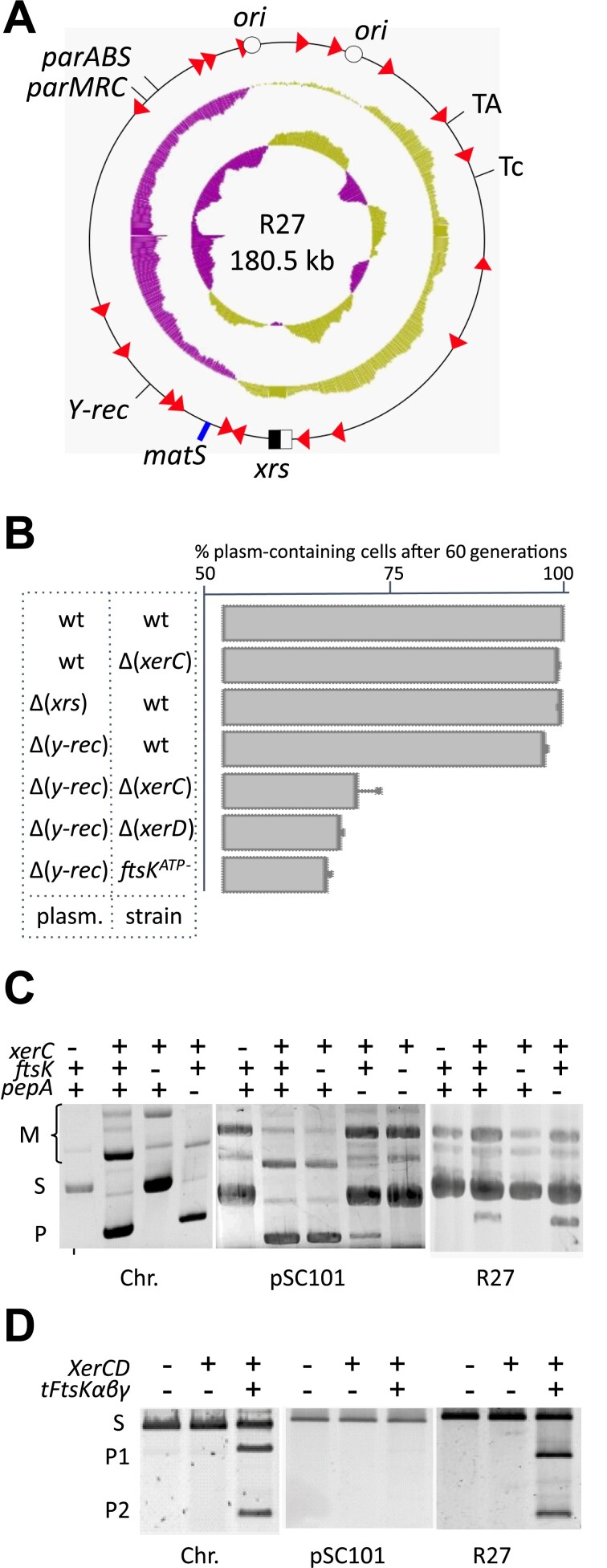
The R27 plasmid resolves dimers in an FtsK-dependent manner. (**A**) Map of R27 showing relevant DNA elements (see Figure 1). The two replication origins (open dots) and the position of genes coding proteins relevant for plasmid maintenance (TA: toxin–antitoxin system; ParABS and ParMRC: partitioning systems; Tc: resistance to tetracycline; Y-rec: site-specific recombinase of the tyrosine family) are indicated. The middle circle shows a plot of the GC skew and the inner circle a plot of the GC content. (**B**) Loss of different R27 derivatives in relevant strains. Relevant genotypes of the plasmids (plasm.) and the strains are indicated. Strains were grown in serial cultures for 60 generations and plated on screening medium. (**C**) Recombination between *xrs*. Plasmids carrying two *xrs* in direct repetition (see Supplementary Figure S2A) were introduced into the indicated strains and grown overnight before gel electrophoresis analysis. Positions of the starting plasmid (S), plasmid with deletion of the fragment separating the two *xrs* (P) and multimeric forms of these two plasmids (M) are indicated. The replicon from which the *xrs* originate in indicated below the gels [*xrs* from the chromosome (*dif*) and from plasmid pSC101 (*psi*) were used as controls]. (**D**) Activation of recombination by FtsK. The same plasmids as in panel (**C**) were incubated with purified XerC and XerD and a variant of FtsK carrying a trimer of the C-terminal domain (tFtsKαβγ) as indicated before analysis by gel electrophoresis. Positions of the starting plasmid (S) and the two deletion products (P1 and P2) are indicated.

We analyzed how R27, a large plasmid (180.5 kb) isolated from pathogenic *Salmonella typhi* (20), resolves dimers. Sequence analysis revealed a toxin–antitoxin system, which we inactivated and replaced by the *lacI* gene to ease stability studies (Figure 3A; see the ‘Material and methods’ section). A GC skew and a KOPS bias ran from replication origins to the opposite region, which contains a *matS* site and an *xrs* devoid of predicted AS. A gene encoding a site-specific recombinase of the Y-recombinase family (*Y-rec*) was also present. Stability assays shown that as the wild-type R27, the *xrs* and the *y-rec* deleted variants of R27 were stably maintained in a *wt* strain (Figure 3B). In contrast, the Δ(*y-rec*) variant was readily lost in strains inactivated for XerC, XerD or the ATPase activity of FtsK.

The R27 *xrs* is 28 bp long and differs from known *xrs* in its XerC binding site (Supplementary Figure S1A). It was less efficiently bound than the *dif* site by XerC in EMSA experiments (Supplementary Figure S1B), although complexes with XerD and with both XerC and XerD formed as efficiently as with *dif*. The R27 *xrs* was inserted with its flanking sequences as direct repetition in a multicopy plasmid to ease recombination studies (see the ‘Material and methods’ section) and the resulting plasmid was used to transform chosen strains (Figure 3C) and for *in vitro* recombination using XerC, XerD and an FtsK variant promoting hexamer formation and DNA translocation (tFtsKαβγ) (Figure 3D). Comparing with equivalent plasmids containing the chromosome-borne *xrs*, *dif*, or the pSC101-borne *xrs*, *psi*, a 28-bp-long *xrs* that mostly recombines under the control of its AS (16,29,31), shows that the R27 *xrs* behaves as *dif* and was not affected by PepA inactivation but by FtsK inactivation. Taken together, our results show that R27 possesses two redundant systems to resolve dimers: a self-encoded Y-Rec system and an *xrs* recombining in an FtsK-dependent manner.

### Large replicons rely on FtsK-mediated control

As for the R27 *xrs*, we cloned *xrs* belonging to chosen replicons of different sizes (Table 1) and assayed their capacity to recombine (see the ‘Material and methods’ section; Table 1). All cloned *xrs* recombined in an XerCD-dependent manner, showing they are active *xrs* (Supplementary Figure S2). Consistent with their association with ASs, plasmid pIGRW12 (5 kb) and R218 (114 kb) *xrs* recombined in a PepA-dependent manner. In contrast, *xrs* cloned from larger replicons, predicted not associated with AS, all recombined in an FtsK-dependent manner. FtsK-induced recombination assayed at a subset of the cloned *xrs* yielded the same conclusion (Table 1). These data validate our prediction of *xrs* and ASs and confirm that the *xrs* found in large secondary replicons recombine in an FtsK-dependent manner.

**Table 1.** Recombination at chosen xrs

Replicon	Size (kb)	Recombination				
		wt	*xerC*	*ftsk*	*pepA*	*In vitro*
chr.	4639	+	−	−	+	+
pRAHAQ01	616	+	−	−	+	ND
pEC-IMPQ	324	+	−	−	ND	+
pCFSAN	294	ND	ND	ND	ND	+
R478	275	+	−	−	ND	+
pAPEC01R	241	ND	ND	ND	ND	+
pEQ1	239	+	−	−	+	+
pHCM1	218	+	−	−	ND	ND
R27	180	+	−	−	+	+
RS218	114	+	−	+	±	ND
pSC101	9	+	−	+	±	−
ColE1	7	+	−	+	−	−
pIGRW12	5	+	−	+	−	−

Recombination was monitored *in vivo* in a wild type (wt) or chosen mutant strains (for *xerC*, *xerD*, *pepA* or *ftsK*) transformed using reporter plasmids carrying a pair of *xrs*, cloned from the indicated replicon, in direct repetition, and *in vitro* using purified XerC, XerD and the tFtsKαβγ variant of FtsK, as in Figure 2C and D, respectively (see the ‘Material and methods’ section). +: Readily detected recombination products; −: no product detected; ±: intermediate activity; ND: not done.

### KOPS biases and *matS* sites appear on large replicons

**Figure 4.**
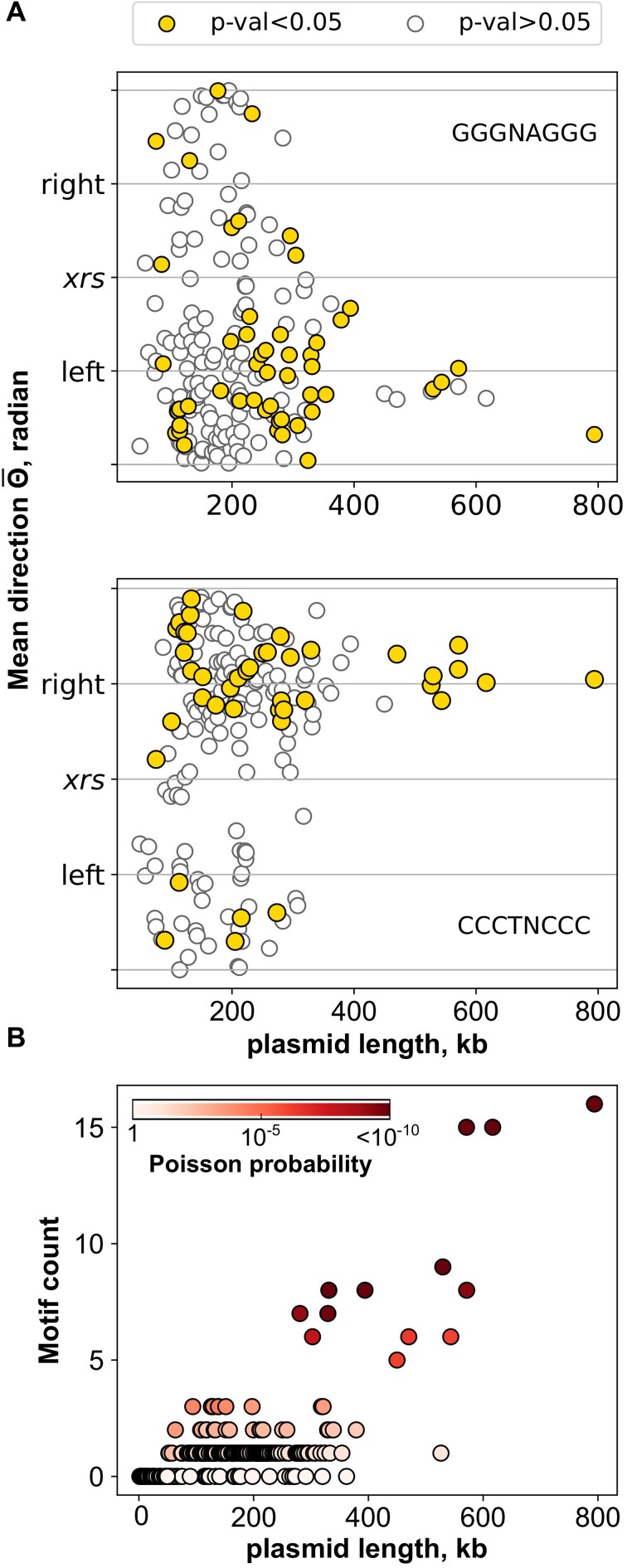
Large replicons tend to adopt a chromosome-like arrangement. (**A**) KOPS biases appear in large replicons. Scatter plots of the mean angle (or direction) of the forward (GGGnAGGG, top) and reverse (CCCTnCCC, bottom) KOPS. Replicons for which the KOPS distribution significantly departs from uniformity (see the ‘Material and methods’ section) are highlighted as filled circles. Other replicons are symbolized by empty circles outlined. Replicons with <3 KOPS were not considered in this analysis. (**B**) *matS* are over-represented in large replicons. Scatter plot of *matS* counts versus plasmid size. Each dot represents one replicon. The intensity of circle filling illustrates the probability of observing such counts in randomized sequences according to a Markov model with a chain length of 4 parameterized for each plasmid (see the ‘Material and methods’ section).

We next searched for KOPS and *matS* DNA sites, two DNA elements participating in the control of FtsK activity in the case of the chromosome (18). KOPS are recognized by FtsK and orient its activity, so that KOPS orientation pointing toward the *xrs* is important whereas KOPS occurrence is less or not important. Consistently, we did not find a significant enrichment of KOPS in replicons harboring *xrs* compared to replicons with no *xrs* (see the ‘Material and methods’ section). To measure KOPS bias, we transformed the position of KOPS and their reverse motif as angles on a circle defined as one strand of the plasmid sequence centered on *xrs* (Figure 4A and Supplementary Figure S3A). The mean angle of the KOPS positions thus points toward the position of the circle where KOPS are most concentrated (see the ‘Material and methods’ section). Figure 4A shows that this mean direction of clustering of the KOPS and reverse KOPS appears to point toward the middle of each halves of the replicon on both sides of the *xrs*. As plasmid size increases, sufficient KOPS were found to calculate significant clustering (41% of plasmids above 75 kb; yellow markers in Figure 4A). Furthermore, when KOPS cluster in one half of the replicon, reverse KOPS tend to cluster in the opposite half, thus placing the *xrs* in the region of converging KOPS orientation (64 of the 78 plasmids with significant KOPS clustering; Supplementary Figure S3B). We conclude that KOPS biases tend to appear in replicons large enough to harbor multiple KOPS and that *xrs* tend to position in KOPS-converging regions.

The *matS* sites are palindromic 13-bp-long sequences degenerated at the three central positions (17). MatP is conserved across the bacteria included in this study, and the residues involved in the interaction with *matS* are identical (Supplementary Figure S3C). Detection of *matS* was performed using a position-specific scoring matrix (see the ‘Material and methods’ section; Supplementary Figure S3D) constructed from the 23 *matS* sites identified on the *E. coli* chromosome (17). Most plasmids contained no *matS*, which was expected from the size of *matS*. Replicons larger than 100 kb tend to contain at least one *matS* and replicons larger than 300 kb contained at least two *matS* with few exceptions (Figure 4B). To determine whether or not *matS* are enriched in these replicons, we measured *matS* occurrence in series of recoded plasmids of the same length and base composition (see the ‘Material and methods’ section), allowing to calculate a Poisson probability of how likely it is to observe by chance the actual number of *matS* in the real plasmid sequence (Figure 4B). We concluded that replicon above 300 kb are significantly enriched in *matS*. Importantly, replicons enriched in *matS* belong to different families, showing that *matS* appearance occurred independently in these different replicons.

## Discussion

A means of dealing with the dimerization of circular replicons is certainly mandatory, as dimers and larger multimers pose a threat to the stable maintenance of replicons, interfering with their segregation and, in some cases, replication (12). For replicons replicating in theta mode, dimer resolution relies on site-specific recombination, which must be controlled so that dimers are better resolved than created. We have shown that the mode of control used primarily depends on replicon size. Replicons larger than 250 kb all contain a Xer recombination site (*xrs*) recombining in an FtsK-dependent manner as the chromosome-borne *xrs*, *dif*. This contrasts with replicons of lower size range, which either use a different control of XerCD/*xrs* recombination in the case of small HCN plasmids or a self-encoded recombination system. We conclude that replicons above 250 kb have to adopt a chromosome-like control of dimer resolution. Consistently, the largest secondary replicons tend to acquire a KOPS-converging zone around their *xrs*, as well as *matS* sites, controlling FtsK and MatP activities, respectively, during segregation of the main chromosome.

The control of XerCD/*xrs* recombination in small plasmids requires DNA slithering over the complete length of the plasmid for the two ASs flanking the *xrs* to get together (Figure 1) (16). Other recombination systems use the same kind of control (32). This mechanism obviously depends on plasmid size and may not be efficient enough in the case of large replicons; for example, the efficiency of recombination by the RES/*res* system of transposon γδ drops two orders of magnitude when the two *res* sites are separated by 100 kb (33). The prevalence of FtsK-mediated control in large replicons may thus be primarily due to the poor efficiency of other kinds of control. The prevalence of 28-bp-long *xrs* devoid of AS in large replicons also suggests that recombination systems other than XerCD/*xrs* do not easily acquire FtsK-mediated control. This may appear surprising as the Cre/*loxP* system from bacteriophage P1 has been shown to substitute functionally to the chromosome *dif* site in an FtsK-dependent manner (29). Consistently, the *loxP* site is located opposite to the replication origin in the plasmidic form of P1 and the P1 genome shows replichore organization from *ori* to *loxP*, with detectable GC skew and KOPS bias (34). However, only chromosome dimer resolution by Cre/*loxP*, not recombination as such, depends on FtsK, allowing the Cre/*loxP* system to play other roles. A strict dependence on FtsK may thus be selected in the case of large replicons and may be easily acquired only in the case of XerCD/*xrs*.

It is generally admitted that chromids originate from plasmids growing in size and acquiring features of the main chromosome, as bases and codon composition and core genes (5,6). This occurred independently in bacterial orders, as judged by the different replication origins and initiator proteins. This evolution includes the adaptation of mechanisms involved in replicon maintenance. When known, chromids have the same copy number per cell as main chromosomes. Their replication is controlled so that termination is synchronous with that of the main chromosome (35–37), pointing to the post-replicative steps, unlinking and partitioning, as the most important to couple with the main chromosome and the cell cycle (12). We previously reported that the partitioning system used by replicons primarily depends on their size (28). In enterobacteria, secondary replicons above 200 kb all possess a ParAB*S* partition system, whereas smaller replicons may have any or no partition system. An equivalent step size applies to the acquisition of FtsK-dependent dimer resolution, coupling the unlinking of sister replicon to cytokinesis in the case of main chromosomes. It thus appears that at least in enterobacteria, the 200–300 kb size range corresponds to a transition above which replicons cannot rely only on plasmid-like maintenance mechanisms and must acquire chromosome-like ones. Consistently, few replicons are in this size range and very few above (Figure 2). We suspect that these replicons are selected for their very low copy number because, in addition to their metabolic load, their size can interfere with the normal dynamics of the main chromosome, as suggested by simulation studies (28). Their low copy number and large size then condition the maintenance required: efficient ParAB*S* system for partitioning and integration of dimer resolution with cell division. Since dimer resolution is integrated by MatP and FtsK in the global segregation pattern of *ter* in the case of the chromosome (18), which includes a functional link with decatenation by topoisomerase IV (38), FtsK-dependent dimer resolution might be accompanied by the integration of the whole unlinking process with cell division.

## Supplementary Material

gkae1300_Supplemental_Files

## Data Availability

Data are available in the supplementary material. The built-in Python package is available at the following sites: https://github.com/python/cpython/tree/3.12/Lib/re, https://doi.org/10.5281/zenodo.14046855, https://src.koda.cnrs.fr/genome-dynamics/Publications/fournes2024.

## Notes

Present address: Florian Fournes, Department of Fundamental Microbiology, Faculty of Biology and Medicine, University of Lausane, Switzerland.

Present address: Jean Cury, Molecular Diversity of Microbes, Institut Pasteur, CNRS, Paris, France.
