# Supplementary material for: The pathway to resolve dimeric forms distinguishes plasmids from megaplasmids in Enterobacteriaceae
Source: Nucleic Acids Res. 2025 Jan 11;53(2):gkae1300. doi: 10.1093/nar/gkae1300 (PMC11724359; doi:10.1093/nar/gkae1300)
Supplement: gkae1300_Supplemental_Files [file gkae1300_supplemental_files.zip › Sup file 1.pdf]

The pathway to resolve dimeric forms distinguishes plasmids from megaplasmids in Enterobacteriaceae.

Florian Fournes, Manuel Campos*, Jean Cury, Caroline Schiavon, Carine Pagès, Marie Touchon, Eduardo PC Rocha, Philippe Rousseau, François Cornet*

* for correspondence : francois.cornet@univ-tlse3.fr ; manuel.campos@univ-tlse3.fr

Supplementary file 1:

- **Supplementary figures S1, S2 and S3**
- **Supplementary text**

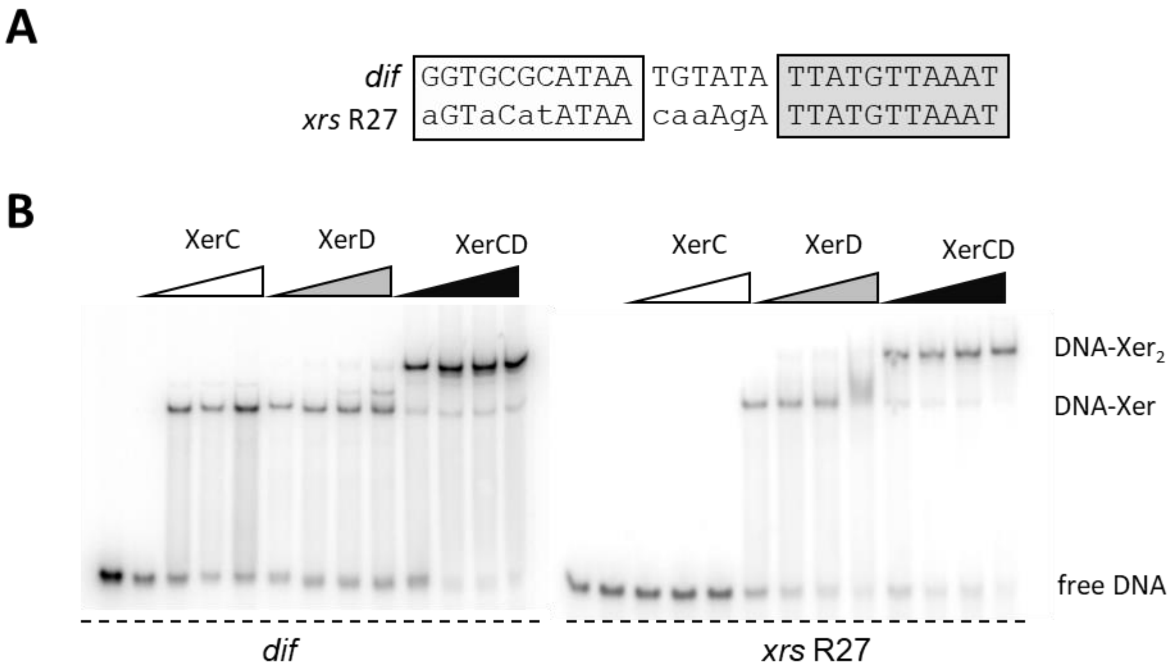

Supplementary figure S1: Interaction between XerCD and the *xrs* of plasmid R27.

A) Sequence comparison between the *dif* site and the *xrs* found on R27 (*xrs44.154* in our nomenclature, see sup file S6). Non-conserved residues are indicated.

B) EMSA of *dif* or the R27 *xrs* by $XerCD_{Ec}$. The DNA fragments are 28bp long and radiolabeled. XerC and XerD ranged from 0.2 mM to 0.8 mM. Free DNA, DNA complexed to one recombinase (DNA-Xer), and DNA complexed to two recombinases (DNA-Xer2) are indicated.

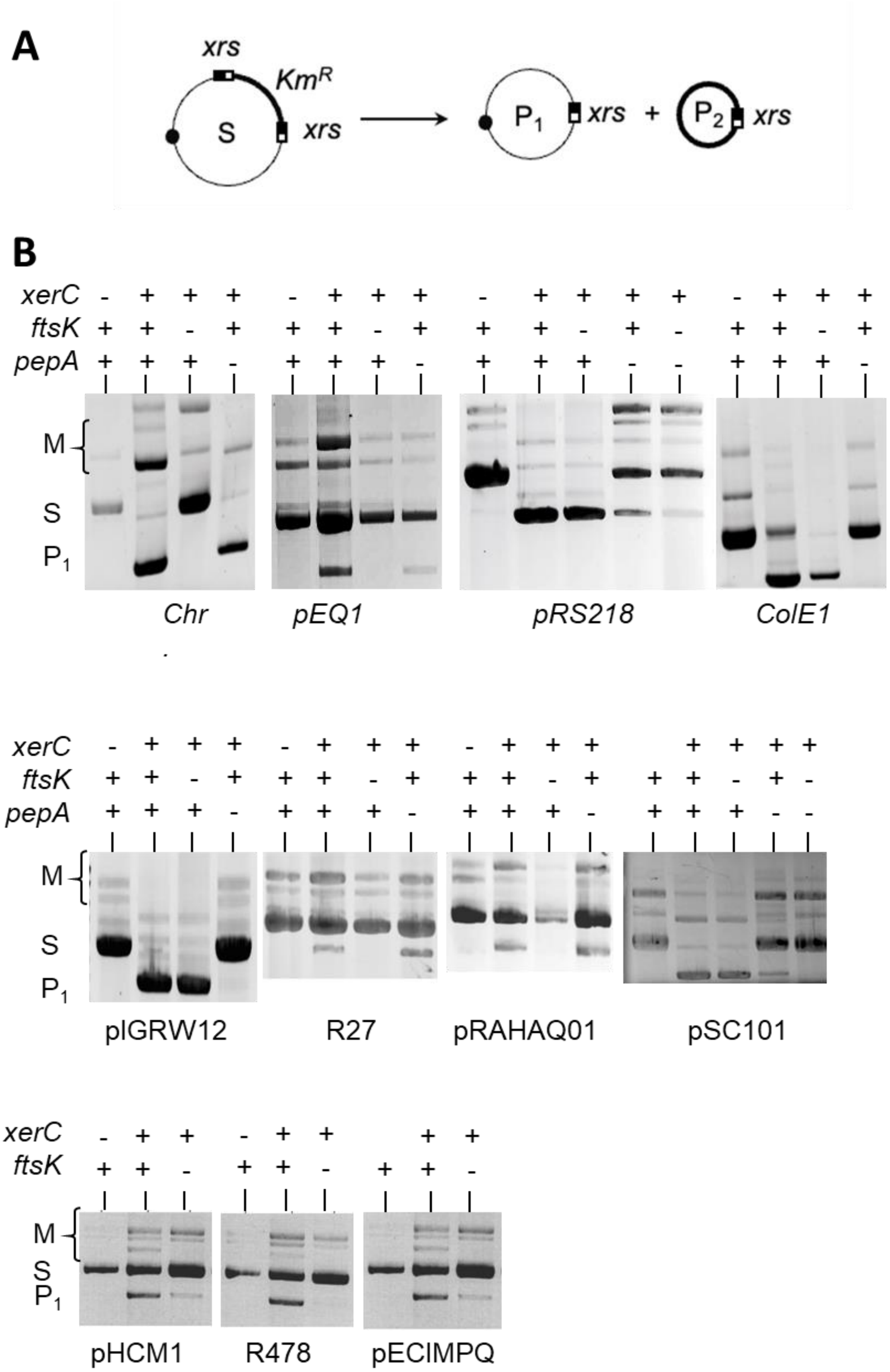
A
xrs
Km^R
S
xrs
P_1
xrs
+
P_2
xrs
B
xerC
ftsK
pepA
M
S
P_1
Chr
pEQ1
pRS218
ColE1
pIGRW12
R27
pRAHAQ01
pSC101
pHCM1
R478
pECIMPQ

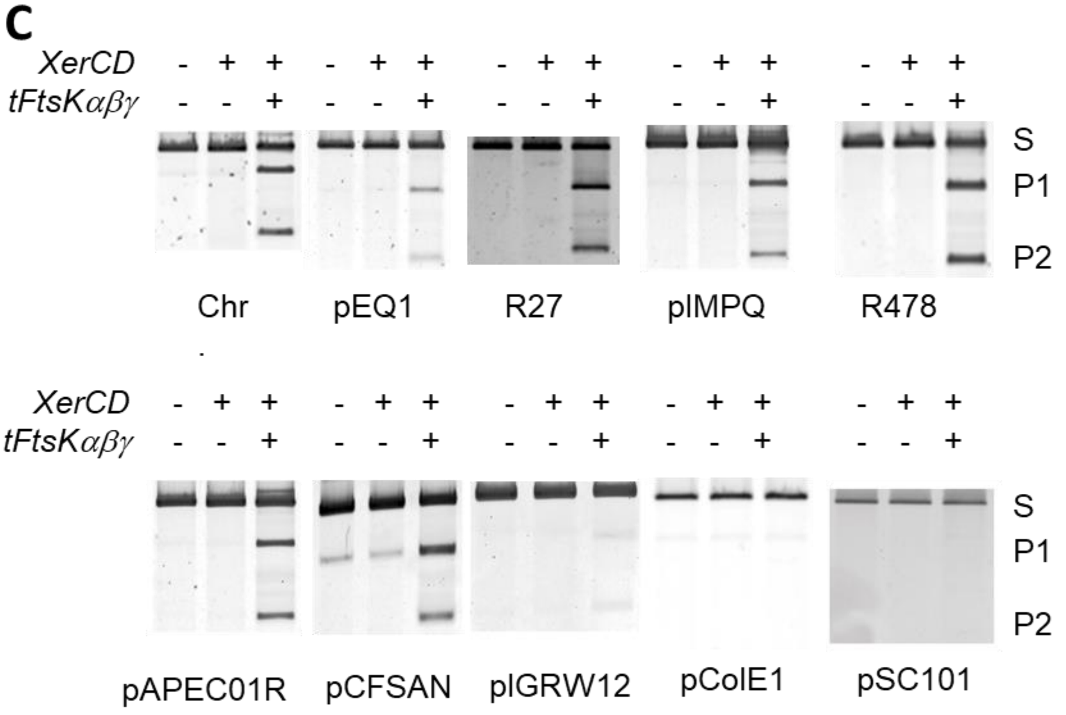

Supplementary figure S2: Recombination at cloned *xrs.*

A) Recombination assay using plasmids carrying directly repeated *xrs*. Recombination deletes the intervening sequence yielding two recombination products (P1 and P2). P2 is not replicative, thus not retrieved in *in vivo* assays.

B) *In vivo* recombination. Plasmids were transformed into the relevant strains carrying the indicated mutation (Methods). Transformants were grown before plasmid extraction and analysis by gel electrophoresis. Position of the starting plasmid (S) and P1 product (P1), and their multimeric forms (M) are indicated.

C) *In vitro* recombination. Plasmids were incubated with XerCD and tFtsKαβγ, a variant of FtsK containing a trimer of the C-terminal domain, as indicated, then analyzed by gel electrophoresis. Positions of the starting plasmid (S) and the P1 and P2 recombination products are indicated.

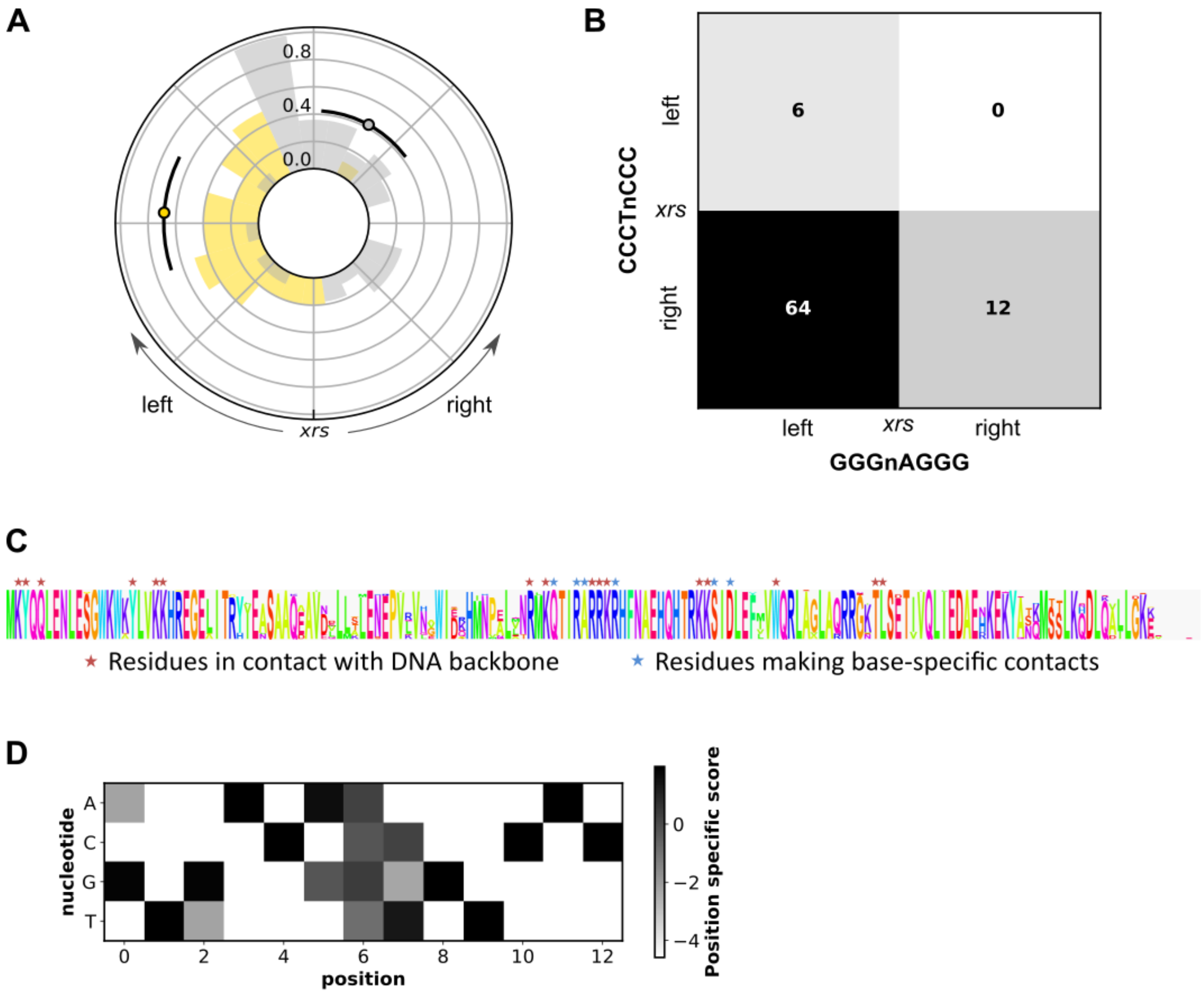

Figure S3: KOPS instances tend to cluster on opposite half of plasmids and *matS* sites are expected to be the same in all bacterial genomes of the collection.

A. Circular histogram of KOPS in their forward (grey bars) and reverse (gold bars) orientation over the sequence of the largest plasmid of the collection (NCBI id: NC_014838). The circular sequence is orientated so that the *xrs* site is situated at the center of the sequence, with a right and left half dominated by the presence of the KOPS motif or its reverse complement, respectively.

B. Contingency table showing the number of plasmids with clustering of the KOPS motif in its forward and reverse orientation in the left or right half of the DNA molecule. Only plasmids with significant clustering of the KOPS motif are considered in this table.

C. Protein alignment of 402 MatP sequences annotated in the 427 unique genomes associated with the 914 plasmids of the collection.

D. Position Specific Scoring Matrix derived from the 23 *matS* sites identified on the chromosome of *E. coli* K12 MG1655 (Mercier et al., 2008).

Supplementary text

xrs previously characterized

Sequences are shown with their XerC-binding site left and their central region isolated (middle 6 to 8 bp). Names of their replicon is given. These *xrs* were used to start the search. Retrieved *xrs* are shown in sup file 1.

GGTGCGCATAA	TGTATA	TTATGTTAAAT	*E. coli* (*dif*)
GGTGCCGACAA	CGGATG	TTATGGTAAAT	ColA
GGTGCGTACAA	CGGGAG	TTATGGTAAAT	ColE3
GGTGCACGCAA	CAGATG	TTATGGTAAAT	pJHCMW1
GGTACCGATAA	GGGATG	TTATGGTAAAT	CloDF13
GGTGCGCGCAA	GATCCA	TTATGTTAAAC	pSC101
GGTGCGCGTAA	TGAGACG	TTATGGTAAAT	NTP16
GGTGCGTACAA	TTAAGGGA	TTATGGTAAAT	ColE1

ArgR binding site – containing accessory sequences (most divergent exemples)

Potential ArgR binding sites are bolded. The seven first ASs were previously characterized and used to start the search.

>**ColE1**, gi|9507253:3708-3938 Plasmid ColE1, complete sequence
ACCCTCTGGT**TGCAT**AGGT**ATTCA**TACGGTTAAAATTTATCAGGCGCGATCGCGCAGTTTTTAGGGTG
GTTTGTTGCCATTTTTACCTGTCTGCTGCCGTGATCGCGCTGAACGCGTTTTAGCGGTGCGTACAATT
AAGGGATTATGGTAAAT
>**ColA**, gi|9507286:1868-2096 Plasmid ColA, complete sequence
TTCACCTGGC**TGCAT**GGTT**ATGCA**GTCGGGTAAAATTTATCAGGTGCGTTCGGGGCGGTTTACCGGGT
GGTTTGTTGCCGGTTTTACCTGTCTTCCGCCCCGAACGCAGTGAACACGTCCGGAGCGGTGCCGACAA
CGGATGTTATGGTAAAT
>**ColE3**, gi|487267:160-371 Escherichia coli plasmid ColE3-CA38 DNA, replicon region encoding Rep protein
ATCGCTTTCG**TGAAT**AGTT**ATGCA**GGCCCCTGAAAACGATTCTGACGCGTTTTTTCGGTTTTGCCTGG
TGTTTTCCTGTCTTTTTGCGTTTTTTGCGTCAGAACGCGTCTGAGGGCGTTTTAAGGGGTGCGTACAA
CGGGAGTTATGGTAAAT
>**CloDF13**, gi|10955253:4773-5001 Escherichia coli K-12 P678-54 plasmid CloDF13, complete sequence
TGCAGTTGCG**TGCAT**AGGC**ATGCA**TTAGGATAAAATTTACCGGGCGCGTTTCCGGCGGTTTTCCGGGT
GGGTTGTTGCTTGTTTTATCCCGTAGCCGCCGGAAACGCCGCCAGTGCCTTACTGGCGGTACCGATAA
GGGATGTTATGGTAAAT
>**NTP16**, gi|9507429:2141-2348 Plasmid NTP16, complete sequence
TTCACTTTCA**TGCAT**AGCT**ATGCA**GTGAGCTGAAAGCGATCCTGACGCATTTTTCCGGTTTACCCCGG
GGAAAACATCTCTTTTTGCGGTGTCTGCGTCAGAATCGCGTTCAGCGCGTTTTGGCGGTGCGCGTAAT
GAGACGTTATGGTAAAT
>**pJHCMW1**, gi|19774233:185-485 Klebsiella pneumoniae plasmid pJHCMW1, complete sequence
TGCACTTGCG**CGCAT**ACTC**ATGCA**TGCCGTAAAAACAGAGCCTGCGCGTTTCTGGCGGGTTTTCGGGT
GGTTTGTTGCCTGTTTTACCGGTTTCCCGTCAGAAACGCCCTGAGGGCCTCTCAGGCGGTGCACGCAA
CAGATGTTATGGTAAAT
>**pIGRW12**, gb|EF088686.1|:2104-2404 Escherichia coli plasmid pIGRW12, complete sequence
TGCATCTGGC**TGCAT**GACT**ATGCA**CCAGTGTAATATTCAGCGGCTGCGTTTCTGGTGTTTTTTCGTTA

TGTCTGTCGCCACTTTCTCGCGTCTGGCACCGGAAACGCAGCCAACCCCGTTTCAGCGGTGCGGGCAA
CGGATGTTATGGTAAAT
>**pAm08CD7339xrs1**, gb|GQ149345.1|:4884-6784 Escherichia coli plasmid pAm08CD7339, complete sequence
TCCATTGCGG**TGCAT**GGCT**ATTCA**TGGCCTCACAATTCATCAGAGGCGTTTTGCGGGATTTTAGGCGG
TAAAGAGGTGCGGTTCTGTGGCTTAGCCGCCCAAAACGCGCGTAGAGCGTCTCTGGCGGTGCGGGAAA
TGAGGCGTTATGGTAAAT
>**pAm08CD7339xrs2**, GQ149345.1:c6784-6457 Escherichia coli plasmid pAm08CD7339, complete sequence
GGTATGTCCG**TGCAT**AGCC**ATGCA**GTCGCGCCAGAATCATCAGGGGCGTTTTTTCTCGATATGGGCAC
AGAAAGGTGTGTGTTTCAGGCCGGAGGCCTCAGAAACGCCCTCAGAGGCTCTGAGGCGGTGCACGCAA
CGGATGTTATGGTAAAT
>**pUMNturkey5_5** gb|JRQA01000059.1|:666-966 Escherichia coli strain UMNturkey5 plasmid pUMNturkey5_5, whole genome shotgun sequence
TTCATTTTCG**TGCAT**GGCT**ATGCA**TACACGCAAAACTTATCAGCGCGATTCCGGGCGGTTTTCGGTGA
GAAGACGGGCTCTTTTGTGGCTCAGGACGGAAAAACGCCCTGAGCGCGTCTGAGCGGTGCGCCCAAGG
GATGTTATGGTAAAT
>**PCN061p1**, gb|CP006637.1|:c1434-1134 Escherichia coli PCN061 plasmid PCN061p1, complete sequence
TTCACTTCTG**TGCAT**AGCC**ATGCA**GGCGCGCAGAAATCATCAGGCGCGTTTTTTCACGATATGGACAG
GGAAAGGTGCCGGAAACGCAGAGGACGCGGCAGAAACGCGCTGAGGGCCTCTCAGGGGGTGCGGGCAA
GGGATGTTATGGTAAAT
>**pNPO1**, gb|KF992024.1|:c1692-1392 Escherichia coli strain W1058 plasmid pNPO1, complete sequence
GGCATTTCCG**TGCAT**AGCC**ATGCA**GACGCGCAGAAATCATCAGGAACGTTTTTCGGGGTTTTCCCCGG
GGAAAGGTGCCGGAAATCGGCATGAGGCCGCAGAAACGCGCTGAGAGCGTCTTAGGCGGTACCGATAA
GGGATGTTATGGTAAAT
>**pAPEC-O78-3**, gb|CP010318.1|:1-76 Escherichia coli strain O78-789 plasmid pAPEC-O78-3, complete sequence
TTCAGTTTCG**CGCAT**AACT**ATGCA**TGAGGTTAAAATTTACCAGGCGCGATCGCGGCAGTTTTTCGGGT
GGTTTGTTGCTGTTTTTACCTGTCTGCTGCCGTGATCGCGCTGAACGCGTTTAAGCGGTACGCGCAAT
GCGACGTTATGGTAAAT
>**pCGB40**, gb|JQ776504.1|:3818-4118 Escherichia coli strain CGB40 plasmid pCGB40, complete sequence
GTCAGTCAGG**TGCAT**GGTT**ATGCA**TGGGGCTGAAAATCACGGTATGCGATTCTGAGCGGTTTTCGGTG
AGAAGACGGGGGTTTTTATGGCTCAGGACGTAAAACCGCCCTGAGCGCGTTTCAGCGGTGCGCGTAAT
GACGCGTTATGGTAAAT
>**pMNCRE44_5**, gb|CP010881.1|:c97299-96999 Escherichia coli strain MNCRE44 plasmid pMNCRE44_5, complete sequence
TTCGGTTGAG**TGCAT**ATCC**ATTCA**TAGGGTAGATTCTTAAGTCGCGTTTCTGGTGTTCATTTTCGGGT
GGTTTGTTACTTGTTTTACCGGGGATATGCCAGAAACGCGCTGAGTCAGTCTGGGCGGTGCGCGTAAT
GCGGCGTTATGGTAAAT
>**pSE11-5**, AP009245.1:1610-1765 Escherichia coli SE11 plasmid pSE11-5 DNA, complete sequence
ACCATCTGGT**TGCAT**AGGT**ATTCAT**GCGGTTAAAATTTATCAGGCGCGATCGCGGTAGCTTTTCGGGT
GATTTGTTGTTGGTTTTGGCTGACTGCCGCCCCGTTCGCGGCGAAGCTGTCCGGGGCGGTGCGGGCAA
CAGATGTTATGGTAAAT
>**p1303_5**, CP009169.1:c4099-3946 Escherichia coli 1303 plasmid p1303_5, complete sequence
TTCAGTTTCG**TGCAT**AGTC**ATGCA**ACGCCCTGAAAACGATCCTGACGCATTTTTCCGGTTTTCCTGGG
GGTAAACATTTCTTTTTGCTGTGCCTGCGTCAGAATCGCGCTCAACGCGTTTTAATGGTGCGTACAAT
TAAGGGATTATGGTAAAT
>**pLST424C-10**, NC_019092.1:688-841 Escherichia coli plasmid

pLST424C-10, complete sequence
TGCACCTGGC**TGCAT**AGCC**ATGCA**TCCGGGTATGATTTATCCGGTGCGTTTCTGGCGGGTTTTCGGGT
GGTTTGTTGCCGCTTTTACCGGTTATCCGTCAGAAACGCGCTGAGTCAGTCTGGGCGGTGCGCGTAAT
GGGACGTTATGGTAAAT

>**pEC278**, AY589571.1:2858-3011 Escherichia coli strain 278B plasmid pEC278, complete sequence
TTCACCCGAC**TGCAT**AGCC**ATGCA**GCCGGGTGAAATTTATCTGGCGCAGAATGCGCGGTTTAGCGGAG
AGTTTGCTGCCGGTTTTACCGGGCTGACGCCGCCATTTGTTCCGAACCCGTCCGGGGCGGTGCTGATA
AGGGATGTTATGGTAAAT

>NC_013954.1:2075-2402 Erwinia pyrifoliae strain Ep1/96 complete plasmid pEp2.6
GGCATTCTGC**TGCAT**AACC**ATGCA**TACAGGCAAAAGCGCCTGAGGGCGTTTCTGACGAATTTTCTCCA
GGAAGACAAGGTTTTTTACGGCTTACGGCTTCAAAGGGCTCTGAGTGCGTCTCAGGCGGTGCGGGCAA
CGGGTGTTATGGTAAAT

>NC_017444.1:1713-1913 Erwinia sp. Ejp617 plasmid pJE05, complete sequence
GTTAGTCCGG**CGCAT**AACG**ATTCA**ACCGCTCAGAACTTACCAGAGCGATTCTGAGCGGTTTTCAGTGG
GGGATATGCGGGAGTTTTGGCGGTTTGACCTCAAAATCGTGCTGAGGGGCTCTCAGGCGGTTCGCGCA
ACGGGTGTTATGGTAAAT

>NZ_CP012166.1:1243-1443 Enterobacter hormaechei subsp. oharae strain 34978 plasmid p34978-2.725kb, complete sequence
GTCATTCTGG**TGCAT**AGTC**ATGCA**TGCCGTTAAAATTTAACAGGAGCGTTTCTGGCGGGTTCCGGGGT
GGTTTGTTGTGGTTTTTGGTCATGGTTCCGTCAGAAAAGCGCTGAGTGCGTCTGAGGCGGTGCGGGCA
ACGGATGTTATGGTAAAT

>NZ_CP016445.1:c2903-2703 Edwardsiella piscicida strain S11-285 plasmid unnamed1, complete sequence
TGCAGTGTCG**TGAAT**GGCT**ATGCA**ACGCCGCCAGAATCATCCTGCGCGATTTTGGCCGTCTTGGGGGT
ACGAAGCTGCGTCTTTTGCCGATAACGCCTCAGAATCGCGCTCAGCGCGTCTGAGGTGGTGCGCCGAG
CGGACGTTATGGTAAAT

>NC_020212.1:c370-170 Serratia marcescens WW4 plasmid pSmWW4, complete sequence
CACGCCGGGT**TGAAT**AACC**ATTCA**CGCTGCCAAACGGCCTGTATGCTATTTTGCGGGCGTTTTAGCGC
AGAAGTGAGGAGGTTTTGTGGCTTCCGCCTCAGAAGCGCGCTGAGGGTGCCTGAGGCGGTGCGGGAAA
CGGATGTTATGGTAAAT

>NZ_CP008845.1:1133-1333 Klebsiella michiganensis strain M1 plasmid pKOXM1D, complete sequence
TGCAGTGATG**TGAAT**AAGTG**ATGCA**TATGCGTAAAATAAATCAGGTGCGTTTCTGGTGGTTTTCGGTG
TACTTGCCACCACCTTTTGCCCGTCAGCCATCAGAAACGCCCTGAGTGCCTCCGGCGCGGTGCGGGTA
AGAAGACGTTATGGTAAAT

>NZ_CP011316.1:c3777-3669 Klebsiella pneumoniae subsp. pneumoniae strain 234-12 plasmid pKpn23412-4, complete sequence
CGCACTTGCG**TGCAT**ACTC**ATGCA**TGACGTGAAAACAGAGCTAGCGTGTTTTTGGCTGATTTTTTGAT
AGTTTGTTGCCTGTTTTACCAGTTTCCCGTCAGAAACACCCTGAGGCCGTTTGGGCGGTGCGTACAAT
TAGGGTGTTATGGTAAAT

>NZ_CP011434.1:1-78 Salmonella enterica subsp. enterica strain YU39 plasmid pYU39_4.2, complete sequence
TTCACTCCTG**TGCAT**AGCC**ATGCA**GACGCGCAGAAATCATCAGCGCGTTTCTGAGCGGTTTTCAGGGG

GTAAGATGTGTTCTTTTGCAGGTGAGGTCGTAGAAACGCCCTGAGAGCCTCTCAGGCGGTGCACGCAA
CAGATGTTATGGTAAAT
>NC_009652.1:1295-1495 Klebsiella pneumoniae subsp. pneumoniae MGH 78578 plasmid pKPN6, complete sequence
GTCAGTCTGG**TGCAT**AACG**ATTCA**TGCCCGTAAAACGCCCTGGAGCGATTTTGAGGCGTTTTCAGGGG
GTAAGACGTAGGATTTAATGGGTTACGCCCCAGAATCGTTCTGAGGCCGTTTTAGCGGTGCGTGTAAT
GACGCGTTATGGTAAAT
>NZ_FO818639.1:7427-7627 Xenorhabdus bovienii str. CS03 plasmid XBC_p, complete genome
CACGGGGGAA**TGCAT**AATT**ATGCA**TTCGTTGAAAAACGGCTGTGCGCGATTTTGCGGGTTTTTGGAGG
GAAAGAGCGTGTGTTTTACCGGGTTGAGGCGAAAACTGCGCTGAATGGCGTACAGGCGGTTCTGGCAA
CGGCTGTTATGTTAAAT
>NZ_CP011627.1:12470-12623 Klebsiella oxytoca strain CAV1374 plasmid pCAV1374-14, complete sequence
TGCGGTGGTG**TGCAT**AGCC**ATGCA**TATGCGTAAAATAAATCAGGTGCGTTTCTGGCGGGTTTTCGGGT
GGTTTGTTGCCTGTTTTACCGGTTTCCTGCCAGAAACGCCCTGAGGCCGTTTTCGCGGTGCGCGTAAA
AAGGCTTTATGTTAAAT
>NZ_CP009366.1:c38191-37991 Yersinia enterocolitica strain WA plasmid, complete sequence
GCAGCCAGGC**TGCAT**AGGT**ATTCA**TGCGGTTAAAATTTATCGGGTGCGATCGCGATAGTTTTTCGGGG
AGTTTGTTGCTCTTTCTGCCTGTTTACTGCTGTGATCGCGCTAAACGCATTTCTGCGGTGCGTTAAAT
CCATCTTATGTTAAAT
>NZ_CP017187.1:c48843-48690 Enterobacter cloacae complex 'Hoffmann cluster III' strain DSM 14563 plasmid pDSMZ14563, complete sequence
AGCACTTTTG**TGCGT**TTGT**ATGCA**TTGAGGTAAAATTCATCTCCTTCGTTTCTGGCGGGTTTGTTGGT
GGGTTGTTGCCTGTTTCACCCGGTTCCTGTCAGAAACGCGCTCCGGCCGTCTGAGCTGTGCGCGTAAT
GAAGCATTATTGTAAAT
>NZ_CP014127.1:c57954-57802 Pantoea agglomerans strain FDAARGOS_160 plasmid unnamed2, complete sequence
GAGCTCTCAC**TGCAT**AGCC**ATGCA**GATGCGCCGAAATCATCAGGAGTATTTTAAGACGTTTAAGCAGA
GAAAGGTGCCGGAAACGGCCCTCAGGCCGCAGAAACGCGCTGAGCGCGCCTGCGGGGGTGAAGGCAAG
CACTGTTATGTTAAAG

ArcA binding site – containing accessory sequences

Potential ArgR binding sites are bolded. The first AS was previously characterized and used to start the search.

>**pSC101**, gi|10955533:6582-6810 Salmonella enterica subsp. enterica serovar Typhimurium plasmid pSC101, complete sequence
CAAACCTGAAGCCGGATCTGCGATTCTG**ATAACAAACT**AGCAACACCAGAACAGCCCGTTTGCGGGCA
GCAAAACCCGTACTTTTGGACGTTCCGGCGGTTTTTTGTGGCGAGTGGTGTTCGGGCGGTGCGCGCAA
GATCCATTATGTTAAAC
>**pECO-b75**, CP009861.1:23-178 Escherichia coli strain ECONIH1 plasmid pECO-b75, complete sequence
ATGAGGCGAAACTGAAAACGAGATTTCA**TTAACAAATA**AGCAACATAAAAAACGGTGGAAAACGCCCA
GGAAAACATGATCTTTTGAGCGGATTTTTTAGATCCGACAATGAACGGTGATCCGGTGGTGCCGATAA

CGTCCATTATGTTAAAT
>NC_010695.1:1400-1556 Erwinia tasmaniensis strain ET1/99 complete plasmid pET09
ACCGGCTGAAACCGGATCCGCGATTCTG**ATAACAAGCC**GGCAACACCAAAACAGCCCGTTTTTGGCCG
GCAACACCCGCGCTTTTGGACGTTCCGGCGGGTTTTGGCGATGAGTGGTGTTGCAGTGGTGCGCGCAA
GAACTCTTATGTTAAAT
>NZ_LN890526.1:29396-29596 Salmonella enterica subsp. enterica serovar Weltevreden genome assembly 99_3134, plasmid : 3
TTGACCTGAAATTAGAAACGACTTTTTA**GTAACAAATT**AGTAACATTAAAAGCTATGCTAAATTACTA
GGTAAAGTTGATCTTTTGAGGTGTTTACTCCGATATTGGTGCCAAAGTGGATCGAGTGGTGCCGATAA
CGACCATTATGTTAAAT
